# From identification to accountability in private equity hospital datasets

**DOI:** 10.1093/haschl/qxag112

**Published:** 2026-05-09

**Authors:** Brett D Cohen

**Affiliations:** Department of Internal Medicine, St.Luke's University Health Network, Bethlehem, PA, USA

**Re:** Kim S, Naslund M, Wang X, et al. A practitioner's guide to using data on private equity hospital acquisitions. Health Aff Sch. 2026;4(4):qxag071.

The Kim et al. dataset is a substantial achievement that lowers the barrier to reproducible research on private equity (PE) hospital acquisitions.^1^ By integrating six commercial deal databases into a validated catalog of 141 deals across 555 short-term acute care hospitals (2000-2024) and releasing the deal list and construction code openly on GitHub, the authors close one of the most durable threats to comparability. Three observations as the dataset moves into use.

First, the paper itself underscores that PE is not a monolithic treatment. LBOs typically involve full operational control and substantial leverage; PIPE and growth equity transactions are minority capital injections with materially different downstream incentives. With LBOs accounting for 71% of hospital-deal observations and PIPE another 20%,^1^ studies that aggregate across deal types risk obscuring real heterogeneity. Aggregation should be a deliberate design choice authors justify, not a default.

Second, the latency problem extends from outcomes to exposure. Studies published in 2025 still rely on Medicare data ending in 2019.^2^ The authors acknowledge their dataset “is not a live, automated feed and will require further manual updating”^1^ and will post any future revisions, though cadence and sustainment are unspecified. A named institutional steward, analogous to the Private Equity Stakeholder Project tracker, would close this gap.

Third, research infrastructure is not accountability infrastructure. The current release is a CSV with stable hospital identifiers, intended for users fluent in AHA-CMS linkage. A lightweight query layer over the existing data could serve three audiences: residency applicants, referring and transferring clinicians, and patients and community boards evaluating local hospital decisions. The January 2025 Prospect Medical Holdings bankruptcy, which closed Taylor Hospital on April 26 and Crozer-Chester Medical Center on May 2,^3^ illustrates the cost of opacity for each. From January through late April, closure remained undetermined: none of the three groups had a firm answer on whether or when the hospitals would close. One staff member put the position plainly: “where are these people supposed to go?”^3^ Crozer's family medicine program, a primary care lifeline for nearly 25 000 Delaware County patients a year, relocated to Penn Medicine Chester County Hospital,^4^ but that outcome was luck, not policy. The community absorbed the loss of its largest hospital system; patients and referring clinicians lost local emergency and inpatient capacity.

The methodologic contribution is real. The next step is extending it to audiences whose decisions cannot wait for the next published study.

## Supplementary Material

qxag112_Supplementary_Data
